# Career Adaptability Research: A Literature Review with Scientific Knowledge Mapping in Web of Science

**DOI:** 10.3390/ijerph17165986

**Published:** 2020-08-18

**Authors:** Huaruo Chen, Tingting Fang, Fan Liu, Liman Pang, Ya Wen, Shi Chen, Xueying Gu

**Affiliations:** 1 School of Education Science, Nanjing Normal University, Nanjing 210046, China; 190601021@stu.njnu.edu.cn (H.C.); 190602093@stu.njnu.edu.cn (F.L.); 190602089@stu.njnu.edu.cn (L.P.); 170601026@stu.njnu.edu.cn (Y.W.); 180601021@stu.njnu.edu.cn (S.C.); 2 Center for Research and Reform in Education, Johns Hopkins University, Baltimore, MA 21286, USA; 3 School of Psychology, Nanjing Normal University, Nanjing 210046, China; 192302037@stu.njnu.edu.cn

**Keywords:** career adaptability, literature review, scientific knowledge mapping, CiteSpace

## Abstract

With the rapid development of society and technology, personal adaptability is becoming more and more important. Learning how to adapt to a changing world is becoming one of the necessary conditions for success. Career adaptability can help individuals to smoothly adapt to changes when coping with their career roles, and maintain their ability to balance their career roles, which will affect their important psychological resources for career development and achieve more meaning in life. In recent years, career adaptability has gradually attracted the attention of researchers. Therefore, in order to explore the main factors, such as research focus, the main researchers, its evolution, and the important results of career adaptability in the last ten years, this study used the scientific knowledge mapping software CiteSpace as a research tool, and select related articles from the Web of Science between 2010 to 2020 under the theme of “career adaptability” for data analysis, which can help future researchers to understand current and future career adaptability research and control the research direction of career adaptability. The results of this research indicate that there are direct or indirect connections between different themes, such as the career adaptability scale, career construction, positive personalities, and so on, but few articles integrate multiple research topics. At the same time, the main researchers, research frontiers and network relationships were also obtained. Based on the above findings, the correlative main concept, theoretical structure, evolution, and research progress of career adaptability in the past ten years are discussed.

## 1. Introduction

Under the influence of COVID-19, many fields around the world, such as education and the economy, have been impacted. Many people argued that they cannot smoothly adapt to the transition from offline work and learning to online, which also leads to researchers’ increased concern about adaptability. Therefore, career adaptability is becoming one of hottest research topic in the field of careers in recent years. Career adaptability originated from the core concept of Super’s career development theory, namely career maturity, which has been constantly updated and revised by researchers (Super & Knasel, 1981) [1]. Some experts proposed “career adaptability” instead of “career maturity” after 1981 (Super & Knasel, 1981; Savickas, 1997) [1,2]. The concept of career adaptability was first proposed by Super, which evolved from another core concept of career development, career maturity. Career adaptability refers to the ability of individuals to adapt to changes smoothly and maintain the balance of their career roles when coping with the transition of their own career roles (Super & Knasel, 1981) [1]. As the work world shifts from stable to fluid, how individuals can improve their career resilience to cope with unpredictable situations and make appropriate adjustments has been explored (Savickas, 1997; Savickas, 2005) [2,3]. Simply put, career adaptability are the resources that can successfully manage individuals’ current and anticipated career transitions (Savickas, 1997; Savickas, 2005) [2,3]. These resources are not the core characteristics of the individual, but exist as a meeting point between humans and the environment, so they are psychosocial (Samuel, 2015) [4]. As an adaptive resource, career adaptability is a self-regulatory ability that one can use to solve unfamiliar, complex, and ill-defined problems arising from developmental career tasks, career transitions, and job trauma (Tolentino et al., 2014) [5]. Career adaptability enables individuals to broaden, improve, and ultimately realize self-concept in professional roles, thus creating a working life, bettering life satisfaction, and building a career framework (Porfeli & Savickas, 2012; Ginevra et al., 2018; Ginevra et al., 2017) [6,7,8].

From the 1950s to the 1990s, Super established career development theory [9], career developmental self-concept theory [10], and life-span life-space career theory [2], all of which became the best interpretations and theoretical models of individual career development at that time (Zhao & Guo, 2010; Guan & Li, 2015) [11,12]. Over time, the theory of career adaptability was continuously revised and developed based on previous studies. Later, Savickas, a representative figure in the study of career adaptability, developed a richer and more connotative and extended career adaptability theory (Savivkas, 1997) [2]. Based on this, a career construction theory from the perspectives of individual constructivism, social constructivism, and postmodernism was proposed and gradually established (Savickas, 2005; Guan & Li, 2015; Savickas, 2002; Savickas & Profeli, 2012) [3,12,13,14]. After that, some researchers initially defined career adaptability as the ability to adapt to job needs or transfer, which can suit individual needs (Pratzner & Ashley, 1985) [15]. With the development of society, different researchers have different definitions of occupational adaptability.

Super and Knasel argued that career adaptability is a state of readiness which is required to cope with tasks that can be predicted by current or future job roles and to adapt to unpredictable work or changes in the work environment (Super & Knasel, 1981) [1]. Savickas modified it to be the individual’s state of readiness for predictable career tasks, the career roles involved, and career problems that are unpredictable in career changes or career situations, which is also a quality that allows for change without much difficulty to conform to the new environment (Savivkas, 1997) [2]. Later, Savickas made a more concise definition and supplement to the concept that was the state of preparation and resources needed to respond to current and anticipated career development tasks, including the attitudes, abilities, and behaviors individuals need to match them with work that suits them, which are psychological resources for managing career change, new tasks, and job trauma (Savickas, 2005; Savickas, 2002) [3,13].

Research aiming at the important potential impact of career adaptability on individuals, career development, and the international community has paid attention to the issue of improving youth career readiness. At the same time, career adaptability has become an important component of international research. Therefore, understanding and mastering the latest development trends of career adaptability is not only helpful for future research, but also for discovering the components of career adaptability that have not been received attention.

### Justification and Objectives

It should be noted that career adaptability should not only be considered in the preparation of study and work, but it should also be considered that career adaptability is an important part of life design and satisfaction (Zhou & Lin, 2016) [16]. On the one hand, at the level of theoretical research, the concept of career adaptability has been established, and relevant theories and models have been published and accepted by researchers. The division of career adaptability into each dimension is increasingly clear (Wilkins et al., 2014) [17]. However, as time goes by, especially in the past decade, it should be stated that the life design paradigm was the most important theoretical framework for career adaptability (Maree & Symington, 2015) [18]. Therefore, in the study of career adaptability, it is very important to understand the latest theoretical research. On the other hand, at the level of empirical research, as an important variable, career adaptability has been applied and practiced in many studies. In the field of education, Chen believed that career adaptability is conducive to the promotion of students’ sustainable development education (Chen et al., 2020) [19]. In terms of family factors, Guan pointed out career-specific parenting behaviors linking parents’ vocational characteristics and children’s career adaptability, which means parental support is positively related to parents’ intrinsic fulfillment values and work–life balance values (Guan et al., 2018) [20]. In the field of work, Spurk’s interindividual study concluded that career adaptability and proactive care behaviors showed positive relations between initial individual levels, intraindividual changes in career adaptability, and proactive care behaviors, pointing to a parallel development (Spurk et al., 2019) [21].

Based on the above researchers’ perspectives, it can be clearly understood that career adaptability emphasizes the interaction between individuals and their living environment, and also need to focus on the non-deterministic problems that individuals faced. Therefore, career adaptability can be regarded as a kind of psychological ability for individuals to maintain a balance these elements when they change their career roles. The ability of career adaptability can be cultivated and developed, which is the result of the interaction between the individual and the environment, and it is also an ability that enables individuals to develop. However, the dimensions of career adaptability research are relatively scattered, and there is no relevant research to systematically sort out career adaptability.

After summarizing the relevant studies, this research reviews and analyzes the international studies on occupational adaptability with the help of CiteSpace and describes the research direction, research hotspots, and representatives of international occupational adaptability. The purpose of this research is to help future researchers to understand the concept, theoretical framework, and other factors of career adaptability, and find out the deficiencies of current research, so as to make more valuable contributions to future research in the field of career adaptability.

## 2. Materials and Methods

### 2.1. Research Design

The research methodology to achieve the formulated objectives was bibliometrics, which can be understood as the branch of scientometrics that analyzes scientific publications. The methodological branch is assumed to be based on the potentialities of scientometrics, which can be defined as the statistical and sociometric analysis of the scientific literature through the use of scientific knowledge mapping and questions related to the processes of searching, recording, analyzing, and predicting the academic literature (Martínez et al., 2015) [22].

More specifically, this research was based on analysis of co-words (Hirsch, 2005) [23] and of various bibliometric indicators and indexes (Cobo et al., 2011) [24]. These data allow for the attainment of a set of maps with nodes that show the performance and the location of sub-domains of the constructs connected to “career adaptability”. In addition, the graphic preparation facilitates the development of the themes of career adaptability in the initially established database (López-Robles et al., 2019) [25].

### 2.2. Procedure

This research followed several procedures:

(1) choice of the database to be analyzed (Web of Science).
(2) determination of the key words to be considered (“career adaptability” or “adaptability”). This research used CiteSpace software as a research tool, and selected related articles for data analysis from the beginning of 2010 to March 2020 in order to explore the research topics and future trends, so that future researchers can understand the current and future career adaptability research from the analysis results, and control the research direction of career adaptability.
(3) elaboration of the search equation (“career adaptability” in the categories of “Education Educational Research”, “Education Scientific Disciplines”, “Psychology Educational”, and “Education Special”).
(4) selection of the search process by bringing together the topic process to report documents that included the concept to be analyzed in the metadata, comprising the title, abstract, and keywords. This action allowed access to a first data report of 39,037 publications. Then, this research set up relevant inclusion/exclusion criteria. In order to meet the inclusion criteria, the article must have had career adaptability as the theme or main variable. This inclusion criterion excluded articles like patents, early access, case reports, data papers, editorials, letters, retracted publications, meetings, unspecified, news, biographies, reference materials, report s, retractions, abstracts, and so on. It is worth noting that, in view of the number of international versions of this article and the language limitations of the researchers, this study mainly included research published in English, Chinese, and a small number of other languages (such as Simplicio, 2014, a report in Portuguese) [26].

**Figure 1:**
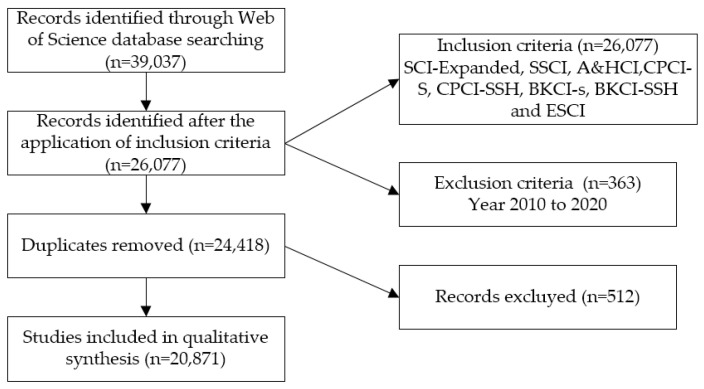
Flowchart according to the PRISMA declaration.

In addition, repeated or improperly indexed documents were deleted. This resulted in a final unit of analysis of 20,871 documents. These actions are reflected in the following flow chart, taking into consideration the protocols of the preferred reporting items for systematic review and meta-analysis protocols (PRISMA-P) matrix (Figure 1).

### 2.3. Software

This research used CiteSpace science knowledge mapping software to conduct a macro analysis of the career adaptability research field. CiteSpace is an information visualization application software for citation analysis written by Dr. Chen from Drexel University, based on the JAVA programming language (Chen et al., 2010) [27]. Because the structure, regularity, and distribution of scientific knowledge are presented by means of visualization, the visualization graphs obtained by such methods are also called “science knowledge mapping”. The maps drawn by CiteSpace software can reveal the knowledge base, hotspot areas, and frontier evolution of the field of scientific knowledge, and enable researchers to intuitively identify the classic basic literature of the corresponding subject area and the evolutionary path of the subject frontier (Chen et al., 2010) [27].

## 3. Results

### 3.1. Author Co-Citation Analysis

**Figure 2:**
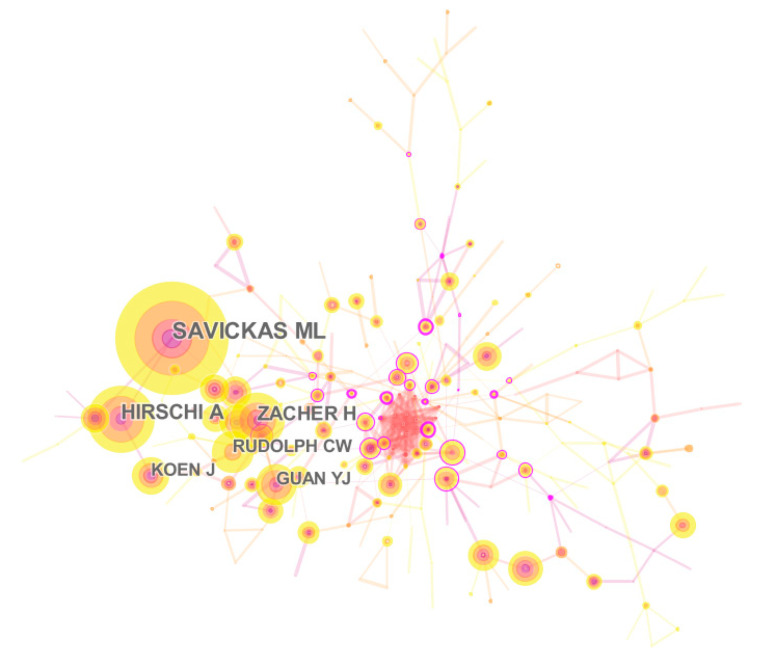
Co-cited atlas of career adaptability authors.

Author co-citation refers to the phenomenon in which two authors are commonly cited by other articles. The co-citation relationship between authors reflects the close relationship between authors in this research direction. The higher the co-citation frequency of two authors, the stronger the correlation between the authors in this academic research direction (Chen et al., 2010) [27]. The CiteSpace visual analysis software was used to carry out author co-citation analysis on the data. When processing the data, cited author was selected as the node type, the year was set to 2010–2020, and other settings kept the default values. After running the data, the authors’ co-cited network map of career adaptability was obtained (see Figure 2). There were 346 network nodes and 739 connections. In the atlas, according to the intermediary centrality of node authors in the co-citation network, representative scholars in the field of career adaptability with greater centrality were shown. At the same time, this research analyzed these highly cited articles.

By sorting out the data from CiteSpace, the co-citation frequency ranking of career adaptability researchers can be obtained (see Table 1). As can be seen from Figure 2 and Table 1, Savickas, Hirschi, Guan, Koen, Zacher, and Rudolph occupied important node positions in the co-cited network. These representative scholars are important figures in the field of career adaptability research. Among them, Savickas is a senior scholar in the field of career development research. Savickas continuously explored the theoretical construction of career adaptability. From the three-dimensional structure model to the four-dimensional structure, Savickas provided an effective method for evaluating the level of individual career adaptability and an important guiding scheme for researchers and career consultants, which had strong practical value (Hartung et al., 2008) [28]. Hirschi established the dimensional structure of the career adaptability questionnaire according to Savickas’s adaptability model. Career adaptability consists of four dimensions, including career decision-making, career planning, career exploration, and career self-confidence. Hirschi developed the youth career adaptability questionnaire and conducted empirical research on the functions and influencing factors of career adaptability (Hirschi, 2009) [29].

A study by Guan et al. [30] mainly took Chinese college students as the research subjects and conducted research on the role of career adaptability of Chinese college graduates in the job-seeking process. Among the four dimensions of career adaptability, career concern and career control were the strongest predictors of job-seeking self-efficacy. In addition, career adaptability also significantly predicted the employment situation and person–environment (P–E) adaptability. These findings carried implications for research on career construction theory, as well as career education and career counseling practices.

Therefore, Koen, Klehe, and Van developed training aimed at providing graduates with career adaptability resources (Koen et al., 2012) [31]. The training included four parts: participants’ self-knowledge, professional environment knowledge, implementation overview, and specific implementation. Koen et al. found that the training successfully enhanced the control and curiosity of college students in the training group by comparing the results of college graduates who received career adaptability training (i.e., the training group) and those who did not receive career adaptability training (i.e., the control group). In addition, among participants who found employment half a year later, training participants reported higher employment quality than did members of the control group. In sum, the results showed that providing graduates with career adaptability resources could raise their chances of finding a qualitatively good job (Koen et al., 2012) [31].

Zacher mainly studied the relationship between career adaptability and personality traits. In the data analysis of a diary survey of enterprise employees, researchers found that openness and conscientiousness could promote career adaptability, while the other three personality types had no significant influence. Meanwhile, Zacher adopted enterprise multi-sample delayed test and situational test methods, which proved that those individuals with high core self-evaluation and concern for the past and the future had relatively high adaptability levels, which confirmed the core view put forward by career construction theory, that is, the development and progress of an individual’s career, fundamentally speaking, involves the realization of individual–environment coordination through the integration of their experience, current situation perception, and future planning (Zacher, 2014) [32].

Rudolph (2017) [33] used meta-analysis to explore career adaptability and adaptation results. One of the studies was based on the career structure model of adaptation and used meta-analysis to examine career adaptability and adaptation measurement, adaptation response, and adaptation relationships. The results supported the significance of career adaptability and related it with personality and other individual difference structures. In terms of adaptation response, the study found that career adaptation was positively correlated with career planning, career exploration, and career decision-making self-efficacy. Career adaptability was not only related to the career success results of subjective evaluation, but also to more objective measures, such as income (Yu et al., 2017) [34]. Career adaptability had a positive effect on subjective well-being (life satisfaction, positive effects, and a low level of negative effects) (Sovet et al., 2018; Santilli et al., 2014; Wilkins et al., 2014) [17,35,36]. Regression analysis clearly showed that some indicators of adaptation outcomes (career and life satisfaction, income, and job performance) were related to, on the one hand, adaptation indicators (such as the Big Five personalities) that were distant (Dalpé et al., 2019) [37]. On the other hand, these adaptation outcome indicators were related to the closer career adaptability indicators, while the more distant adaptation indicators were controlled (Ocampo et al., 2018) [38]. In addition, the indirect effects of these adaptation indicators on the adaptation results were analyzed through career adaptability. That study provided theoretical guidance for researchers seeking to develop intensive research projects on the role of adaptive capacity in career development and professional behavior (Briscoe et al., 2006) [39].

### 3.2. Keyword Co-Occurrence Analysis

**Figure 3:**
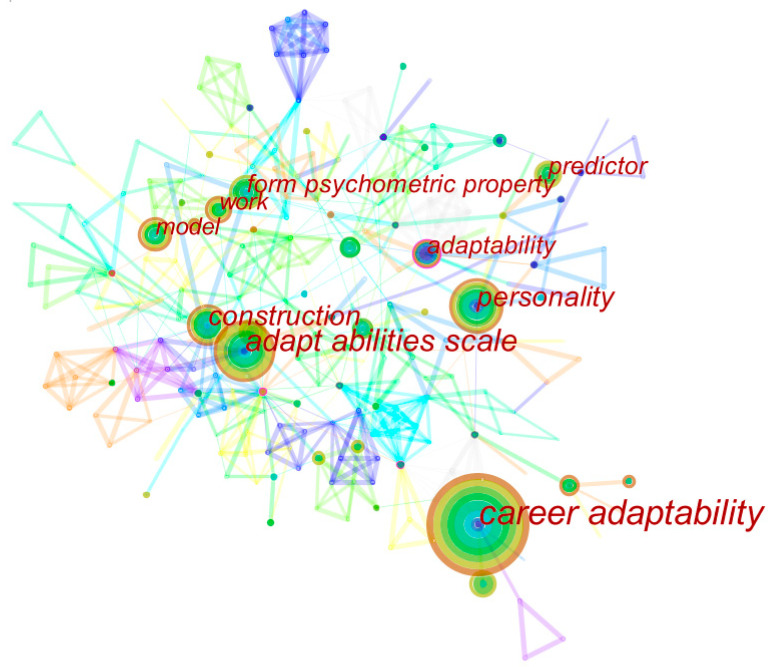
Co-occurrence map of keywords in career adaptability.

Keywords are the extraction of the main content of a paper. Analyzing the keywords can reveal some hot topics in career adaptability research. In this study, CiteSpace was used to analyze the keywords of career adaptability articles. Article data were imported into CiteSpace software. Keyword was selected as the node type. Time slicing was set as 2010–2020, and the value of time partition of year per slice was set as 1. Selection criteria maintained the default value of 50 and the imported data were run to obtain the co-occurrence map of career adaptability research keywords (see Figure 3). After sorting out the data from CiteSpace, 43 high-frequency keywords for career adaptability research were obtained (see Table 2). In a later chapter, this research also classifies these high-frequency keywords into the dimensions of career adaptability.

In the keyword co-occurrence map in Figure 3, the size of each circle refers to the frequency of keyword occurrence, and the larger the circle, the bigger the number of keyword occurrences. Table 2 shows the ranking of keywords derived from the data. The ranking of keywords indicates the ranking of research hotspots in this field. The higher the keywords are ranked, the more often this word is used in this field, which represents a problem to which everyone pays more attention. Judging from the ranking of keywords in Table 2, the top five keywords are “Career Adaptability”, “Adaptability Scale”, “Personality”, “Construction” and “Form Psychological Property”. These keywords have had a relatively high frequency in the past decade. These are hot topics for researchers in this field, who have mainly focused on the issues of career adaptability and scale revision, the relationship with personality, structure establishment, career adaptability, and psychometric attributes.

### 3.3. Research Frontier Topics

**Figure 4:**
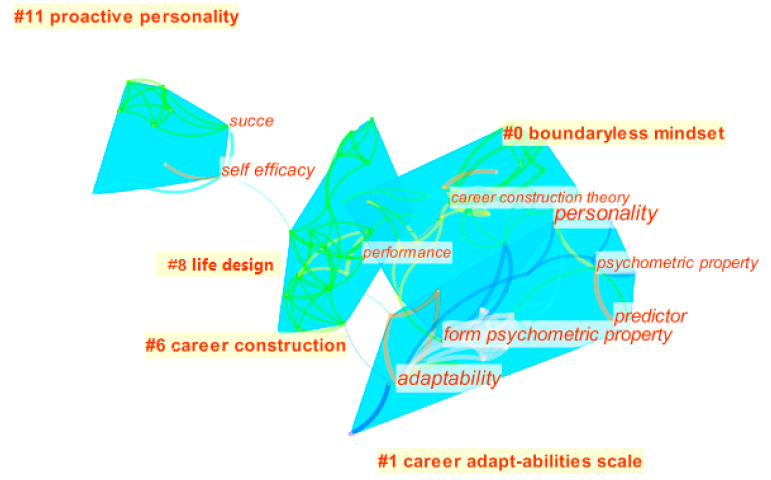
Career adaptability research frontier topics map.

On the basis of keyword processing, cluster view was selected for the visualization of layout to obtain the career adaptability research frontier topics map (see Figure 4), in which the modular Q value (modularity Q = 0.6364) and mean silhouette score (mean silhouette = 0.7465) indicate that the clustering is reasonable. From the map, five clusters automatically identified by CiteSpace can be seen, which represent five frontier topics of career adaptability research. The map shows the basic trend of the research and development of career adaptability in the past 10 years (see Figure 4). The research found that the field includes five main clusters (described by the clustering features of high-frequency keywords): boundaryless mindset, career adaptability scale, career construction, proactive personality, and life design. It is worth noting that there are direct or indirect connections between different topics. For example, a boundaryless mindset in workplace life often encounters a dilemma. A favorable, unfavorable, or neutral attitude may have adverse consequences, which is reflected in the discussion of career adaptability. In order to measure a person’s level of career adaptability, the scale of career adaptability has attracted more and more attention. Career construction theory consists of three main components: life theme, professional personality, and career adaptability. Paying attention to the development of career adaptability will certainly extend to the study of career construction to ensure the integrity of an individual’s career and the classification of professional personalities includes proactive personalities. With the maturity of social development in recent years, people are no longer just satisfied with career success, and more and more researchers found that a great number of people will pay attention to career success and self-satisfaction. Therefore, they gradually design their own complete life to achieve the best level of life satisfaction. Life design comes naturally and has been widely used in recent years. On the other hand, in addition to proactive people, the other four clustering topics are closely linked. This shows that there is an unbalanced relationship between career adaptability topics. In this research, the development and analysis of these five topics will be discussed.

#### 3.3.1. Boundaryless Mindset

The boundaryless mindset refers to a tendency of employees belonging to an organization to pursue cooperation with individuals or organizations outside the organization and actively adapt to the content of cross-border work. The concept of the boundaryless mindset originated from the study of the borderless career. In the past two decades, the research on careers has gradually formed two popular perspectives, one is the protean career, and the other is the boundaryless career. Briscoe and Hall (2006) developed the Boundaryless Career Attitude Scale on the basis of defining relevant concepts, and for the first time explained the concept of boundaryless mindset, namely, “Thus a person with a decidedly high ‘boundaryless’ attitude toward working relationships across organizational boundaries is comfortable, even enthusiastic about creating and sustaining active relationships beyond organizational boundaries” (Briscoe et al., 2006) [39]. The main meaning of the boundaryless mindset is that individuals like and pursue cross-departmental or cross-organizational cooperative relationships, challenge and update their knowledge systems and work abilities, and are positively adaptable to new environments. It is a kind of value judgment of employees in the career field, so as to guide and influence employee behavior, and it further had an effect on organizational development (Ali et al., 2016) [40]. With the deepening of research, researchers also continuously found that career adaptability was related to the boundaryless mindset. For example, in the study of Chan et al., the relationship between career adaptability and the boundaryless mindset was closer (Chan et al., 2015) [41].

#### 3.3.2. Career Construction Theory

Career construction theory was put forward by Savickas, a senior scholar in the field of career counseling practice and research in America and a rising star in the field of Western vocational psychology over the past 20 years (Nota et al., 2014) [42]. Based on the research of Super’s career development theory, Savickas moved from career development to career construction by way of career narration (Walker et al., 2019) [9]. Savickas (2002) proposed 16 exploratory propositions based on the theory of career construction (Savickas, 2002) [13]. Later, inspired by McAdans’s general framework of personality, Savickas merged and developed personal environment fit theory and life theme theory, and further refined those propositions into three aspects of career construction theory (Savickas, 2013) [43]. The traits of different individuals are differential. The tasks and coping strategies faced by individuals in different career stages are developmental. Career development is a dynamic process (Savickas, 2005) [3]. As a result, career construction theory responds to the “what”, “how”, and “why” in individual professional behaviors with vocational personality types, career adaptability, and life themes (Savickas, 2005, 2013) [3,43].

#### 3.3.3. Proactive Personality

The proactive personality first appeared in an article entitled *The Proactive Component of Organization Behavior*, published by Bateman and Crant in *the Journal of Organization Behavior* in 1993 (Bateman & Crant, 1993) [44]. In this study, according to the definition of positive psychology, the concept of the proactive personality was put forward, and it further defined the difference between the active personality and passive personality, whereby individuals with proactive personalities are relatively free from environmental constraints, which thus affect environmental changes. A proactive personality could help people to identify opportunities, take actions, and persevere until they succeed. However, passive individuals show adaptation to the environment and are easily shaped by the environment. They cannot identify and seize the opportunity to change things, seldom show enthusiasm, and rely on the strength of others. Later, other scholars defined the concept of the proactive personality from different perspectives. Greguras and Diefendorff (2010) proposed that individuals with a proactive personality show self-reliance and self-improvement and are mostly future-oriented (Greguras & Diefendorff, 2010) [45]. Parker, Bindl, and Strauss (2010) proposed that the goal of employees with a proactive personality is to continuously improve the work process and results (Parker et al., 2010) [46]. Campbell (2000) summed up the five core characteristics or qualities. Firstly, they can be competent in their own work, showing a high level of professional organization, problem-solving ability, and excellent performance. Secondly, they have a high level of interpersonal competence, leadership, and trustworthiness. Thirdly, they show high level of commitment to organizational goals and a strong sense of responsibility for organizational success, with values consistent with the organization and a positive working attitude. Fourthly, they have positive and enterprising qualities, such as initiative, independent judgement, a high level of engagement and involvement, and the courage to express their ideas. Fifthly, they show the quality of integrity and pursue higher values (Campbell, 2000) [47].

In recent years, the active personality, work performance, and career success are more common. Pan (2018) found that the quality of internships had become an important regulator of the relationship between the active personality and career adaptability, as well as employment success (Pan et al., 2018) [48]. When the quality of internships was low, the indirect influence of the proactive personality on employment success through career adaptability was greater. Jiang (2017) pointed out that the initiative personality promoted personal work development and thus improved career adaptability. Moreover, it was found that the impact of vigorous development on career adaptability was stronger for those with a less proactive personality (Jiang, 2017) [49]. In addition, there are studies on the active personality and leadership. For example, Crant and Bateman explored the relationship between the proactive personality and charismatic leadership. One hundred and fifty-six managers and their directed supervisors participated in the study. The results showed that the higher the managers’ self-report scores for proactive personality, the higher the superiors’ evaluation for charismatic leadership. The results also showed that the variance explained by having a proactive personality was larger than that explained by many additional variables. The variance explained by the social approval of behavior in the role of five personality factors was larger (Bateman, 2000) [50].

#### 3.3.4. Career Adaptability Scale

The Career adaptability scale is the most important research topic in this field. Researchers have developed different scales according to their own research. Therefore, this study has organized the areer adaptability scale across a wider application scope.

(1) Savickas and Porfeli’s Career Maturity Scale

The Career Maturity Scale (CMI-C), as a concise, reliable, and effective measure of career choice readiness, is mainly used in school populations of grade 12 and below. CMI-C has a total of 24 items and consists of five parts, namely: the total score of career choice readiness, the scores of three scales reflecting the adaptability dimensions of attention, curiosity, and self-confidence, and the scores reflecting the relationship style that informs career choice (Savickas & Porfeli, 2011) [51].

(2) Savickas and Others’ Career Adjustment Strength Table

Savickas proposed a four-dimensional construction of career adaptability, which includes four dimensions of career attention, career control, career curiosity, and career confidence and a four-dimensional structure for the career adapt ability scale (CAAS) based on the theoretical model (Savickas & Porfeli, 2012) [14]. The scale has been tested in 13 countries and regions including Europe, America, Japan, and China. It has passed the consistency test under different cultural backgrounds and has high applicability. Subsequently, researchers from many countries and regions have revised the scale in combination with the local culture and have compiled a career adaptability scale more suitable for the region. The scale consists of 24 items and is divided into four dimensions: career attention, career confidence, career curiosity, and career control. Each dimension has six items (Savickas & Porfeli, 2012) [14].

(3) Savickas’ Career Adapt abilities Scale—Short Form (CAAS-SF)

The CAAS-SF is a psychological measurement tool for measuring adaptive resources. The CAAS-SF is a short version of only 12 items based on the CAAS. The development of the CAAS-SF aims to promote the implementation of the CAAS in the research of more different groups and professional backgrounds, reduce the management time for professional consulting and life design practitioners, and promote all aspects of their practical work. The reduction in the number of items also retains the excellent psychometric characteristics of the tool. This scale is composed of four dimensions, and each dimension is evaluated by three items, namely, a job satisfaction survey, general work stress scale, and organizational self-efficacy scale (Maggiori, Rossier, & Savickas, 2017) [52].

(4) Hirschi et al.’s Adolescent Career Adaptability Questionnaire

Hirschi revised the “Adolescent Career Adaptability Questionnaire” dimension structure, which is based on Savickas’s adaptability model and consists of four dimensions: career decision-making, career planning, career exploration, and career confidence (Hirschi, 2009) [29]. In this questionnaire, the career decision-making component is taken from the Career Maturity Inventory (Seifert, 1986) [53] and consists of 12 items. The career planning component is taken from the Career Development Inventory (Seifert, 1985) [54], with 22 items in total. Career exploration consists of two parts, one of which is taken from the Career Development Inventory, with a total of 26 items. The second part is taken from the Career Exploration Survey Scale (Stumpf, Colarelli, & Hartman, 1983) [55] and the Career Exploration Questionnaire (Kracke, 2002) [56], which has 10 questions, including four questions for self-exploration and six questions for environmental exploration. Career confidence is measured by two career ability belief questionnaires. Among them, one questionnaire has three items and the second questionnaire has five items (Hirschi, 2009) [29].

(5) Rottinghaus et al.’s Career Futures Inventory—Revised (CFI-R)

Rottinghaus adopted a rational method to formulate the Career Future Inventory (CFI). The list consists of three subscales: career adaptability (CA), career optimism (CO), and perceived job market knowledge (PK). The scale has a total of 25 items, of which CA has 11 items, CO has 11 items and PK has 3 items (Rottinghaus et al., 2005) [57]. After that, Rottinghaus revised the CFI (Rottinghaus et al., 2012) [58]. The revised CFI (CFI-R) has 28 items, including five internally consistent sub-scales: career agency, career awareness, support, work–life balance, and negative career outlook. The CFI-R evaluates various aspects of career adaptability, including positive career planning attitude, general outcome expectation, Parsons’s tripartite model, and Bandura’s personal agency. The convergent validity and discriminate validity of the CFI-R sub-scale are related to career decision-making, difficulties, self-efficacy, personality optimism, and coping styles. The CFI-R can help individuals understand the current attitude towards various career changes and improve the effectiveness of career counseling by solving the concerns of individuals in the changing career world (Rottinghaus et al., 2012) [58].

(6) Nota et al.’s Career and Work Adaptability Questionnaire (CWAQ)

Over the past 10 years, the aim of the presented studies was to develop a specific, new instrument, “Career and Work Adaptability”, to assess the degree of adaptability in adolescents planning their futures. In 2012, Nota, Ginevra, and Soresi conducted three studies, the first of which aimed to formulate the instrument’s items and to verify its factor structure. The second study confirmed the instrument’s multidimensional structure and evaluated its discriminant validity. The third study was conducted to verify the factorial structure’s cross-gender invariance and to evaluate its stability over time. The results showed that the instrument is an effective and multidimensional instrument for accurately measuring career adaptability. Specifically, it can serve as a useful vocational guidance tool in analyzing adolescents’ career adaptability (Nota et al., 2012) [59].

#### 3.3.5. Life Design

Life design, a new paradigm, was implicit within the constructivist and narrative methods for career intervention that emerged in the 21st century (Savickas, 2012) [60]. Special attention should be paid to the fact that career adaptability has been recorded as a key self-regulation process in career construction and life design in the 21st century (Wen et al., 2020) [61]. However, some studies found that after life design intervention, career adaptability did not changed (Savickas & Porfeli, 2012; Maree et al., 2018b; Maree, 2019) [14,62,63]. Only a few studies found that life design could improve career adaptability or some dimensions (Nota et al., 2016; DaSilva et al., 2020) [64,65].

Life design plays an important role in career adaptability. However, it cannot be neglected that life design still faces challenges (Nota & Rossier, 2015) [66]. In order to solve those challenges, some experts came up with the following paths, which can be considered in future research. One is that career adaptability is an important variable affecting an individual’s career, and life design intervention could be designed around career adaptability, such as increasing intervention time, etc. The other is that similar groups may have common characteristics, so future studies could focus on the common needs of clients, and create more research groups based on life design (DaSilva et al., 2020; Maree & Symington, 2015c) [18,65]. In addition, a more personalized life design could be developed by creatively combining adaptability methods, such as career collages, career portfolios, etc. (Barclay et al., 2019; Ginevra et al., 2018; Ginevra et al., 2016) [67,68,69].

### 3.4. Research Hotspot Evolution

**Figure 5:**
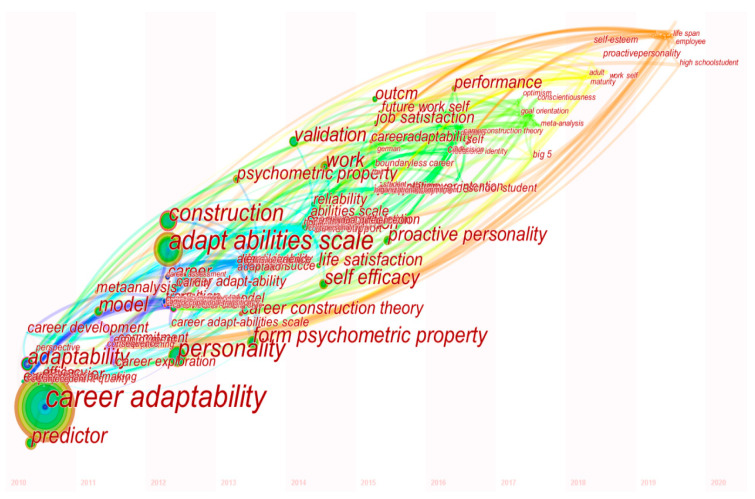
Network map of hotspots in career adaptability research.

Research hotspots refer to topics or issues that are of common interest in a set of articles that have large number of high-frequency occurrences and have internal links within a certain period of time in a scientific field (Chen, 2010) [27]. Keywords are the embodiment of the content of the article, so the analysis of keywords can show the evolution of the hotspots in the research of career adaptability. After importing the data into CiteSpace, “keyword” was selected as the node type in the function selection area, “2010–2020” was selected for the year range. The other settings were left as their default values. In the running edit box, timezone view was selected in layout visualization, and then the map was adjusted to get the research hotspot evolution map of career adaptability (see Figure 5). By objectively showing the dynamics of the research hotspots of career adaptability over time, it can be used to not only grasp the development context of career adaptability research in the time dimension, but also understand the distribution characteristics of the research keywords in different time zones more intuitively.

Through the analysis of the literature and charts (see Figure 5 and Table 3), from the perspective of time distribution, the 2010 study used “predictor” as the hotspot to predict the predictive effects of variables related to career adaptability. For example, Koen et al. adopted a two-wave design to predict the job search strategy and reemployment quality of the unemployed through their career adaptability (Koen et al., 2010) [70]. Duffy studied 1991 undergraduate students and found that the sense of personal control is one of the predictors of career adaptability (Duffy, 2010) [71]. From 2011 to 2013, research mainly focused on the revision, localization, and verification of the career adaptability scale. For example, Savickas revised the CMI (career maturity) (Savickas & Porfeli, 2011) [51]. It was noteworthy that in 2012, localization studied with the CAAS appeared in various regions. In 2013, research mainly verified the effectiveness of the CAAS in different cultural backgrounds. In 2014, studies focused on research related to career adaptability in different occupational groups. For example, Tolentino targeted business students to study the relationship between career adaptability and individual entrepreneurial intentions (Tolentino et al., 2014) [5]. Studies have found that, when individuals formed their entrepreneurial intention, they relied on the adaptive resources of their career adaptability and entrepreneurial self-efficacy, and individuals exposed to family businesses had stronger career adaptability (Tolentino et al., 2014) [5]. Based on career construction theory, Guo’s research examined individual and contextual predictors for the professional competence of Chinese undergraduates majoring in social work and found that career concern and career curiosity predicted social work students’ professional competence, with these relations mediated by the calling in social work (Guo et al., 2014) [72]. In 2015, studies mainly discussed the influence of the proactive personality on career adaptability. Based on Career Construction Theory and Self-Verification Theory, Cai’s research examined the mediating and moderating models for the relations among self-esteem, the proactive personality, career exploration, future work self, and career adaptability and found that both self-esteem and a proactive personality (measured at time 1) positively predicted the future work self and career adaptability (measured at time 2), with these relationships mediated by career exploration (Cai et al., 2015) [73]. Chan found that relationships between the CMI-C and CAAS with entrepreneurial, professional, and leadership career motivation profiles showed that the CAAS was more strongly related to the boundaryless mindset and protean career attitudes, while the CMI-C appeared to relate to more traditional (professional and leadership) career motivations (Chan et al., 2015) [41].

From 2016 to 2020, the research keywords in this field tended to describe diversity, reflected in the diversity of research subjects, including research on different groups of students, refugees, corporate employees, and immigrant groups. For example, Zacher used a quantitative daily diary study design and discovered that daily job demands, daily job autonomy, daily conscientiousness, and daily openness to experience, as well as daily past and future temporal focus, positively predicted daily career adaptability (Zacher, 2016) [74]. Presbitero and Quita found that the career adaptability of overseas immigrants has a positive correlation with their overseas working intention (Presbitero & Quita, 2017) [75]. Obschonka and Wehrle studied the career adaptability of refugee groups (Obschonka et al., 2018; Wehrle et al., 2019) [76,77]. At the same time, there were also studies on career adaptability in China, Turkey, Switzerland, Italy, and other regions during this period, which reflected the diversity of research areas in this field. For example, Saido conducted a qualitative analysis of the role of career adaptation in Japan in career change (Saido & Yoshida, 2016) [78], and Santilli used adolescents from Switzerland and Italy as samples to explore the relationship between career adaptability and life satisfaction (Santilli et al., 2017) [79]. Ozdemir used a qualitative study of the adaptability of the Turkish youth community (Karacan-Ozdemir, 2019) [80]. In addition, from 2016 to 2020, there were many small research hotspots in this research area, mainly around individual factors and situational factors influencing career adaptability research. The research on individual factors mainly focuses on personality traits, highlighting the Big Five and the proactive personality. Rudolph, Lavigne, and Zacher found that each dimension in the Big Five personalities was significantly related to career resilience through a meta-analysis test (Rudolph et al., 2017) [33]. Jiang found that a proactive personality first enabled individuals to thrive at work, which in turn led to improved career adaptability (Jiang, 2017) [49]. The study of contextual factors is mainly focused on social support. For example, Guan et al. found that the level of support from organizations can affect individual career construction to a considerable extent (Guan et al., 2016) [81]. Guan et al. studied the moderating role of traditionality beliefs in the indirect relationships among parental support, career decision-making self-efficacy, and career adaptability among Chinese university students, and found that parental support was associated positively with career decision-making self-efficacy and career adaptability (Guan et al., 2016) [82]. Atac, Dirik, and Tetik found that perceived social support positively predicted career adaptability and that perceived social support plays a moderating role in the relationship between perceptions of self-esteem and career adaptability sub-scales (Atac et al., 2018) [83].

## 4. Discussion

### 4.1. Theory Construction

Super was one of the earliest researchers to discuss the construction of career adaptability theory. In the early days, Super proposed that the measurement of career adaptability should include the following areas: work values and work salience, autonomy or sense of agency, planning or future perspective, exploration and establishment, decision-making, and reflection on experience (Super & Knasel, 1979) [84]. Later, Super revised the construction of adult career adaptability, and proposed a more complete “model of adult career adaptability”, which had five dimensions, including planning, exploration, information, decision-making, and reality orientation. However, the object scope of the career adaptability model constructed by Super was aimed at adults (Super & Knasel, 1981) [1].

In order to make up for this deficiency, Savickas further enriched and improved the theoretical construction of career adaptability based on Super. Initially, Savickas proposed that career adaptability consists of three important dimensions: planful attitudes, self and environmental exploration, and adaptive decisions (Savickas, 1997) [2]. In 2005, Savickas further revised and improved the theoretical construction of career adaptability and proposed a more complete construction model. Savickas believed that the development of individual career adaptability develops along four dimensions or stages, which are career concern, career control, career curiosity, and career confidence (Savickas, 2005) [3]. In the theory of career adaptability, career concern is regarded as the first and most important dimension, which addresses the question “do I have a future?” It means that an individual can pay attention to his/her own future career (Savickas, 2005) [3]. Career control is the second important dimension of career adaptability. It addresses the question of “who owns my future?” that is, the belief that individuals are self-determined and responsible for building their own careers (Savickas, 2005) [3]. Career curiosity reflects the individual’s curiosity attitude, which motivates individuals to explore more careers, and enables teenagers to more realistically explore education and career choices, and then achieve future goals. The basic function of career curiosity in career construction is the same as the function of self-exploration and career exploration in career development theory, which means that individuals are willing to actively try to explore themselves and the work world (Savickas, 2005) [3]. Career self-confidence refers to individuals’ confidence in their problem-solving abilities and self-efficacy beliefs, which can help the individual to build a perfect future and overcome difficulties (Savickas, 2005) [3].

### 4.2. Scale Compilation

In the field of research on career adaptability, the career adaptability scale is undoubtedly an important research topic. Scholars in various countries have also localized the scales of Super and Savickas in light of their own cultural background and actual situation, and with further research in this field, more and more kinds of scales have been developed.

In the process of compiling various career adaptability scales, this study found that most of the subjects of the scales are adults. For example, the career adaptability scales of Savicks, Hirschi, and Rottinghaus. However, there are not many scales specifically designed for high school students and below, such as the Savickas and Porfeli Career Maturity Scale (Savickas & Porfeli, 2011) [51]. In China, secondary vocational students enter jobs after their secondary vocational studies are completed. As a special group, is the adult career adaptation strength chart applicable and is it also applicable to the measurement of the career adaptability of secondary vocational student groups? This needs to be verified by further research.

### 4.3. Influence Factors

When reviewing the literature of this period again, this study found that the factors affecting career adaptability were mainly concentrated in two aspects: one was the variables related to the individual, such as gender, grade (age), personality traits, etc. In terms of gender, Rottinghaus found that there was no significant gender difference in career adaptability among college students (Rottinghaus et al., 2005) [57], and Hirschi also believed that gender did not affect the development of career adaptability among middle school students (Hirschi, 2009) [29]. In terms of age level, most studies believed that grade (age) level was a good predictor of career development, but Hirschi found that grade does not affect the development of career adaptability (Hirschi, 2009) [29]. Therefore, the influence of grade (age) on career adaptability remains to be further studied. As for personality traits, Hirschi focused on the Big Five personality traits and the proactive personality. The second was the variables related to the environment, such as family, social support, and so on. In terms of family factors, the impact of family socioeconomic status and parents on career adaptability was mainly explored. Many scholars believe that family socio-economic status is positively related to the development of career adaptability, and that parenting styles also have an impact on youth career development (Chen et al., 2020; Dietrich & Kracek, 2009) [19,85]. In terms of social support, the social support of students was mainly reflected in family, school, and peers. While the social support of the adult group mainly came from the unit, supervisor, or related policies and measures.

## 5. Conclusions

Based on the scientometrics methodology, the thesis on career adaptability in the Web of Science database was systematized by using CiteSpace, and the science knowledge mapping of career adaptability and its evolution law were described. The results of this research are as follows:

(1) In the field of career adaptability research, authors such as Savickas, Hirschi, Guan, Koen, Zacher, and Rudolph ranked high in the citation index. Among them, Savickas made an important contribution to the theory construction and scale compilation of career adaptability. Other scholars have also made contributions to this field in their research direction.
(2) There are five hot topics in career adaptation research, namely: the boundaryless mindset, career construction, the proactive personality, career adaptability scale, and life design.
(3) The research hotspot evolution in career adaptability is as follows: the keywords of the research hotspots before 2016 were relatively isolated, and research in 2010 focused on the predictive role of variables related to career adaptability with “predictor” as the hotspot. From 2011 to 2013, research was mainly focused on the revision, localization, and verification of the career adaptability scale. A study in 2014 focused on research related to career adaptability in different occupational groups. A study in 2015 mainly discussed the impact of the proactive personality on career adaptability. The keywords of research hotspots after 2016 tended to be diversified, which was reflected in the diversity of influencing factors related to the research object, research area, and career adaptability.

## 6. Limitation

This research has some limitations. Firstly, the types of data that can be processed by the software were limited. This study only selected literature from the Web of Science (WoS) database as the data source. Future research can further enrich the data source. Secondly, this study only discussed the trends and characteristics of international literature research from 2010 to 2020. Future research can also be combined with domestic research for further analysis and comparison. Thirdly, due to the limitation of language, it was impossible for this study to cover all the studies. Finally, when combing the literature, it was found that culture also had an impact on individuals’ career adaptability. Is there any difference in the adaptability of different cultural groups? Researchers in the field of careers can strengthen international cooperation to explore the differences of career adaptability among different cultural groups.

## 7. Future Trends

In a word, according to the above data analysis results, it is not difficult to find that in the past ten years, more and more studies have focused on the practice of career adaptability, and have studied and explored the impact of career adaptability on work and learning even in other areas. Therefore, the main points that this research found, which need to be paid attention to in future research, are summarized as follows.

(1) The diversity of research objects. In the early research of career adaptability, the main focus was on individual working and learning. However, from 2016 to 2020, the research subjects in this field were diverse, including research on different groups of students, refugees, enterprise employees, and migrant groups. Therefore, future research may pay more attention to the research groups that have not received much attention, such as the adaptation of female workers to the workplace, and the maladjustment of diverse populations caused by the current epidemic situation.
(2) The diversity of research tools. The development and use of the career adaptation scale has become mature. However, with the rapid development of society, many factors that affect career adaptability cannot be found only by scales. In the choice of future research tools, qualitative research should be done, such as some field research or role experience. Researchers should research the environment in more depth, and experience the impact of various factors on the research subjects.
(3) The diversity of research relationships. In the current relationship research, career adaptability has been verified in many dimensions, but with more focus on the psychological level. Therefore, future research should not only focus on the relationship between these dimensions and career adaptability, but also pay more attention to the mediating role of career adaptability. In addition, more influencing factors in observational research should also be found. Starting from the data analysis, the relationship between the two and whether they have a positive effect or a negative effect should be explored.

## Figures and Tables

**Table 1:** List of cited frequencies of career adaptability research authors (top six).

Number	Cited Times	Year	Author
1	116	2016	Mark L. Savickas
2	70	2016	Andreas Hirschi
3	57	2016	Hannes Zacher
4	44	2018	Cort W. Rudolph
5	44	2016	Guan Yanjun
6	40	2016	Jessie Koen

**Table 2:** Ranking of 43 high-frequency keywords for career adaptability.

Number	Keyword	Frequency
1	Career Adaptability	108
2	Adaptabilities Scale	55
3	Personality	39
4	Construction	34
5	Form Psychometric Property	23
6	Predictor	23
7	Adaptability	22
8	Work	22
9	Model	20
10	Self-Efficacy	19
11	Validation	18
12	Proactive Personality	16
13	Success	15
14	Psychometric Property	14
15	Satisfaction	14
16	Construct	12
17	Life Satisfaction	11
18	Outcome	11
19	Performance	11
20	Exploration	10
21	Reliability	10
22	Career	9
23	Job Satisfaction	9
24	Transition	9
25	Behavior	8
26	Life	8
27	Career Adaptability	7
28	Career Construction Theory	7
29	Career Adaptability	7
30	Efficacy	7
31	Future Work Self	7
32	Meta-analysis	7
33	Adolescent	6
34	Job Performance	6
35	Five Factor Model	5
36	Abilities Scale	5
37	Development	5
38	Hope	5
39	Job	5
40	School Student	5
41	Self	5
42	Social Support	5
43	Turnover Intention	5
Total	-	649

**Table 3:** Top 10 keywords in career adaptability studies.

Rank	Time	Keywords	Count	Centrality
1	2011	Career Adaptability	108	0.1
2	2013	Adaptabilities Scale	55	0.3
3	2012	Personality	39	0.11
4	2013	Construction	34	0.09
5	2014	Form Psychometric Property	23	0.15
6	2010	Predictor	23	0.03
7	2012	Adaptability	22	0.16
8	2014	Work	22	0.11
9	2014	Model	20	0.1
10	2014	Self-Efficacy	19	0.04
